# Interoception in pregnancy: Implications for peripartum depression

**DOI:** 10.1016/j.neubiorev.2024.105874

**Published:** 2024-09-05

**Authors:** Paul W. Savoca, Laura M. Glynn, Molly M. Fox, Misty C. Richards, Bridget L. Callaghan

**Affiliations:** a Department of Psychology, University of California, Los Angeles, USA; b Department of Psychology, Chapman University, USA; c Department of Anthropology, University of California, Los Angeles, USA; d Department of Psychiatry and Biobehavioral Sciences, University of California, Los Angeles, USA; e David Geffen School of Medicine, University of California, Los Angeles, CA, USA

**Keywords:** Interoception, Early-life adversity, Maternal mental health, Pregnancy, Peripartum depression

## 1. Introduction

Pregnancy is a compressed period of dramatic developmental change and plasticity that occurs at least once in the lives of up to 80 % of adult females in the United States (Carlin and Alfirevic, 2008; Glynn et al., 2018; Schock et al., 2016; Servin-Barthet et al., 2023; Soma-Pillay et al., 2016). Such changes include hormonal flux, growth of the placental organ, immunological and commensal microbial shifts, and a rapidly changing body to accommodate the growing fetus (Davis and Narayan, 2020; Glynn et al., 2018; Kazma et al., 2020). Less appreciated, and documented, is the development and remodeling of brain tissue that occurs during pregnancy and across the early postpartum period, some aspects of which, subsequently, stay with the mother throughout her life (Hoekzema et al., 2017; Kinsley et al., 1999, 2008; Lambert and Kinsley, 2012; Luders et al., 2022; Pawluski et al., 2022). Together, this physiological and neurological flexibility enables the mother to meet the demands of pregnancy, allowing for the successful gestation of the fetus, as well as the preparation of the mother for birth, breastfeeding, and parenting. While such changes represent adaptations, this is an often-fraught period of life – pregnancy and parenting can be rewarding, while at the same time posing mental health challenges for some (Bennett et al., 2004; Stuart-Parrigon and Stuart, 2014).

Depression is a serious complication of pregnancy, historically occurring in at least 11 % of pregnant women and 13 % of new mothers, although in recent years both of these rates have been estimated to be closer to 20 % (Bennett et al., 2004; Dagher et al., 2021; Gaynes et al., 2005; Jahan et al., 2021; Liu et al., 2022). In part, the rates of peripartum depression were observed to be higher during and soon after the onset of the COVID-19 pandemic (Safi-Keykaleh et al., 2022). Peripartum depression poses risks not only for the mother, but also for the fetus/child (Meaney, 2018; Sawyer et al., 2019). As such, finding ways to address peripartum depression is a public health priority. Despite the high rates of the illness, little is known about the mechanisms that shift the adaptive plasticity of pregnancy toward greater incidence of depressive symptoms and affective pathologies.

One mechanism implicated in adult depression outside of pregnancy is interoception – the process by which the brain perceives, integrates, and models sensory information generated from within the body (Craig, 2002; Khalsa et al., 2018; Sherrington, 1952). The prevailing theory is that impaired interoceptive signaling can prevent the brain from making accurate predictions of the body’s energetic needs, causing metabolic inefficiencies inherent to depressive illnesses (Barrett et al., 2016; Quigley et al., 2021). Indeed, in non-pregnant adults, impaired interoception appears to be a risk factor for developing depressive symptoms (Avery et al., 2014; Brand et al., 2023; Dunne et al., 2021; Eggart et al., 2019). Given the metabolic demands of pregnancy, interoceptive deficits are a potential mechanism for peripartum (the period during and shortly following pregnancy) depression illnesses.

Although few studies have examined interoception links with peripartum depression, some studies have documented that pregnancy is associated with greater self-reported interoceptive functioning (Crossland et al., 2022; Noda et al., 2022; Singh Solorzano et al., 2022). This may serve as an adaptive function to cope with the increased metabolic demands of this stage of development. In turn, we suggest that one pathway to resilience against peripartum depression likely involves improvements in interoception during pregnancy. Specifically, improved interoception during pregnancy should mitigate increased metabolic demand, and conversely, risk for peripartum depression involves insufficient improvements in interoception, such that there is a mismatch with the increased metabolic demands of this period of life. Crucially, we propose that known vulnerability factors for peripartum depression (such as exposure to early-life adversity) may influence an individual’s interoceptive processing away from the resilience pathway and towards the risk pathway. Thus, understanding how interoception changes during pregnancy may be critical to understanding peripartum depression illnesses.

In this perspective piece, we propose a novel mechanistic framework for peripartum depression in which improved interoceptive functioning in pregnancy and postpartum typically protects against depressive symptoms, but when there is a failure to improve interoception, the result is an inability to maintain metabolic efficiency, which ultimately poses risk for depression. First, we will review an emerging theory that links the modeling and predictive regulation of metabolic resources as central to the expression of many depressive symptoms. Then, focusing on the period of pregnancy to articulate the framework, we will discuss the physiological changes induced by pregnancy that place an increased metabolic demand on the pregnant mother. Following, we will address the neural and physiological changes of pregnancy that may enable enhanced interoception during this period of life. We will then review factors (e.g., childhood adversity) that may modulate pregnancy related changes in interoception. To demonstrate how the framework just described in pregnancy extends to the postpartum period, we will then discuss how the unique neurobiology and psychological features of the postpartum period, relative to during pregnancy, act to maintain high metabolic demands on the mother, as well as impact interoception in ways that can lead to resilience or risk for postpartum depression. We end by highlighting potential treatment targets suggested by our framework.

## 2. Interoception, allostasis, and depression

Work from the field of evolutionary neuroscience suggests a primary role of the brain is to maintain an efficient energetic state of the body via predictive regulation – a process termed allostasis (Schulkin and Sterling, 2019; Sterling, 2012; Sterling and Laughlin, 2015; Table 1). This process is supported by interoception which allows us to predict and sense (both consciously and non-consciously) the internal state of the body and mount metabolic actions to maintain efficient energy expenditure. To be a “good regulator” of the body, one must generate an accurate model of the body (Conant and Ashby, 1970). This suggests that precise interoceptive modeling of the body is critical to efficient allostasis (Quigley et al., 2021; Sennesh et al., 2022; Shaffer et al., 2023). Interoception includes both the unconscious modeling of the body, of primary importance to supporting allostasis, and also the conscious perception and integration of internal sensory information (Craig, 2002; Khalsa et al., 2018; Sherrington, 1952). As the state of the body is not directly accessible to the brain, to generate a model of the body, the brain must try to infer the state of the body using internal sensory (i.e., interoceptive) information. The more accurate interoceptive information is, the better the brain can model the state of the body and then select actions (both skeleto-motor and viscera-motor) which maximize metabolic efficiency (Sennesh et al., 2022). However, if either the interoceptive information used to make the model, or the model itself, are inaccurate, then so too will be the selected actions, resulting in poor metabolic efficiency.

Emerging theory and empirical research points to widespread metabolic inefficiencies as a key feature which may underlie many depression symptoms and major depressive disorder (Allen et al., 2018; Barrett et al., 2016; Chan et al., 2019). In other words, these theories center metabolic deficits as a defining feature of depressive illnesses. From an evolutionary perspective, many of the symptoms and characteristics of depression are proposed to serve an adaptive function to conserve limited energetic resources (Andrews and Durisko, 2017). Indeed, many of the major symptoms commonly associated with depression (e.g., fatigue, persistent negative affect, anhedonia, etc.) can be directly attributed to a compromised energetic state of the body (Shaffer et al., 2022). In the dominant theory conceptualizing depression as a metabolic disorder (Barrett et al., 2016; Shaffer et al., 2022), the internal model used to maintain allostasis does not update to match the current state of the body, that is, the internal model becomes insensitive to prediction errors, termed a “locked-in” internal model. In turn, the brain becomes unable to manage and estimate metabolic resources accurately, and the internal model fails to efficiently update, leading to metabolic inefficiencies.

Use of an example – glucose regulation, which is positively associated with interoceptive ability (Young et al., 2019) – can facilitate an understanding of how allostasis and interoception interact though an *active inference* framework (Barrett, 2017; Friston et al., 2017; Seth, 2013). The process begins with a prediction (i.e., *prior*; Friston, 2010) about the current blood-glucose level. Importantly, this prediction incorporates past-experience (e.g., previous glucose levels), current internal context (e.g., basal metabolic rate), and current external context (e.g., time of day), to make the predicted state physiologically adaptive for the current goal of the body (Hesp et al., 2021; Sajid et al., 2021). At the same time, sensory surfaces within the periphery relay interoceptive information about the current glucose levels to the brain. As prediction signals are relayed from the brain to the body and afferent sensory signals are relayed from the body to the brain, these signals meet and are compared to each other (Barrett and Simmons, 2015). When the predicted glucose level matches the sensed glucose level, the prediction is confirmed, and no further action is required. However, when the predicted and sensed levels do not match, the difference is encoded as a *prediction error* (Friston, 2005). These prediction errors are then fed-forward to the brain regions from which the predictions were generated (Hutchinson and Barrett, 2019).

In a state of prediction error, the brain has two options: 1) change the *action*, 2) change the *inference* (Friston et al., 2017). A change in *action* might include signaling the pancreas to release insulin to reduce glucose levels. A change in *inference* would involve updating expectations for future predictions (i.e., predicting higher glucose levels in the future). Importantly, these options are not mutually exclusive, but instead typically work in tandem to maximize efficiency (i.e., minimize prediction error). These options generally map onto allostasis and interoception – allostasis is the action (i.e., controlling the body via skeleto- and viscera-motor predictions) whereas interoception is the inference (i. e., updating interoceptive priors/predictions or enhancing the precision of signaling; Sennesh et al., 2022). Critically, greater precision and accuracy of interoception can both lead to more adaptive interoceptive predictions and ultimately more efficient allostasis.

In terms of subjective experience, interoceptive and allostatic signaling are particularly important for informing the experience of affect (Feldman et al., 2024). Affect is our low-dimensional feeling state, composed of two components, valence and arousal, which can be used to construct our more abstract psychological phenomena such as emotions and mood (Barrett, 2017; Barrett and Russell, 1999). Actions that move our body toward an optimal allostatic state are likely experienced as positive valence, where actions that move us away from an optimal state are experienced as negative valence (Keramati and Gutkin, 2014). Therefore, in states of high allostatic prediction errors, we are likely to subjectively experience high levels of negatively valanced affect. In addition, interoceptive signals are likely very relevant for informing our experience of arousal – e.g., conveying information about our heart-rate, blood pressure etc. (Quigley et al., 2021). Thus, allostatic prediction errors and interoceptive information likely work together to inform both the valence and arousal dimensions of affective experiences.

Importantly, because of the unprecedented physiological changes associated with pregnancy, this stage of life may challenge the brain to generate precise estimates of the state of the body. In a healthy, normative pregnancy, a woman’s brain would successfully perceive the major changes to metabolic demand, update predictions in accordance with the metabolic challenge, and thus adjust. However, in the pathological circumstance that the pregnant woman’s brain does not successfully update its model of metabolic demands, depression can occur. The fact that metabolic efficiency is relevant to the functioning of the whole body also helps to explain why depression, both in pregnant and non-pregnant adults, is associated with alterations in a wide range of biological systems including the brain, immune, and endocrine systems (Aruldass et al., 2021; Dowlati et al., 2010; Juruena, 2014; Lamers et al., 2013; Zeng et al., 2012). As such, while the symptoms of depression may be adaptive to reduce energy expenditure in the short term, the behaviors become maladaptive when the internal model fails to efficiently update, as predictions (which drive actions) are no longer based on an accurate representation of the state of the body.

### 2.1. Neural underpinnings of interoception

**Fig. 1.**
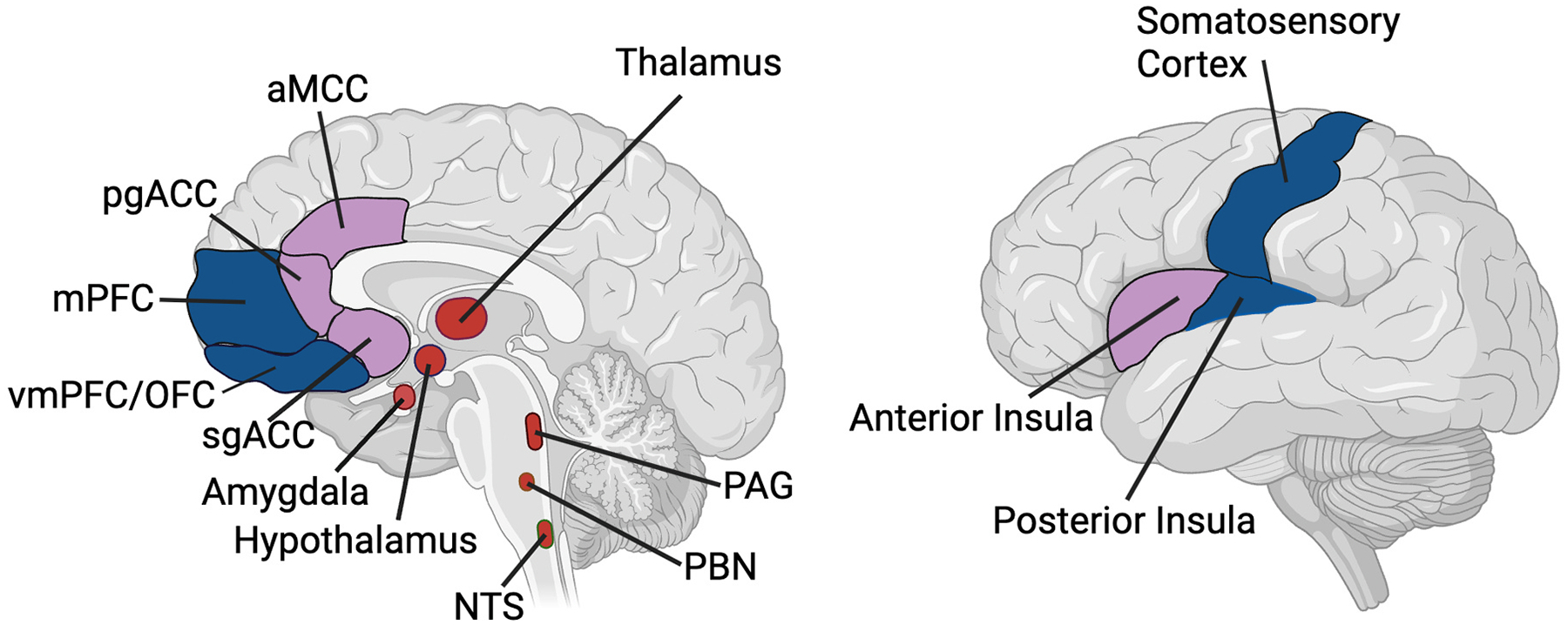
Neural regions that support interoceptive functioning. Regions that have relatively more granular laminar structure are depicted in dark blue, while regions with relatively more agranular structure are depicted in light purple. The structural organization exists on a spectrum, but for simplicity we dicotomized regions into relatively more granular or relatively more agranular. Subcortical regions involved in interoception are colored red. Abbreviations: Anterior Midcingulate (aMCC); Pregengual Anterior Cingulate Cortex (pgACC); Medial Prefrontal Cortex (mPFC); Ventromedial/Orbitofrontal Prefrontal Cortex (vmPFC/OFC); Subgenual Anterior Cingulate Cortex (sgACC), Nucleus of the Solitary Tract (NTS), Parabrachial Nucleus (PBN), and Periaqueductal Gray (PAG).

Several brain regions and networks have been reliably associated with interoceptive functioning. Initially, and often of primary focus, the insula has been identified as the neural region most critical for interoception (Critchley et al., 2004). Within the insula, the dorsal posterior insula (dpIns) has been considered the primary interoceptive cortex (i.e., receiving interoceptive information from the body via the brainstem) while the anterior insula (aIns) has been hypothesized to support more abstract interoceptive processes (such as conscious awareness of bodily sensations; Barrett and Simmons, 2015; Berntson and Khalsa, 2021; Craig, 2009; Craig, 2002; Khalsa et al., 2009; Fig. 1).

Despite the intense focus of interoception research on the insula, growing evidence supports the view that interoception occurs via a distributed network of neural activity that is dynamic and synchronized (Kleckner et al., 2017; Zhang et al., 2023). This network includes activity within the insula, as well as the subgenual anterior cingulate cortex (sgACC), pregenual anterior cingulate cortex (pgACC), anterior midcingulate cortex (aMCC), and other default mode and salience network regions such as medial prefrontal cortex (mPFC) and orbitofrontal cortex (OFC). There is also evidence that some interoceptive information can be processed in the primary somatosensory (S1) cortex (Khalsa et al., 2009). Additionally, several subcortical regions are involved in the relay and processing of interoceptive information, including the nucleus of the solitary tract (NTS), parabrachial nucleus (PBN), periaqueductal gray (PAG), thalamus, hypothalamus and amygdala (Berntson and Khalsa, 2021; Chen et al., 2021). Together this distributed network is thought to be critical for the central processing of interoceptive information (Fig. 1).

From an active inference standpoint, the flow of interoceptive information (i.e., interoceptive predictions and prediction errors) is thought to follow a cyto-architectural gradient of the brain (Barbas, 2015; Bastos et al., 2012; Hutchinson and Barrett, 2019; Katsumi et al., 2021). This gradient is primarily determined by the laminar structure (i. e., the organization of neurons within layers of a cortical column) of the cortex. Granular regions are characterized by six well-distinguished laminar layers and tend to be close to sensory (interoceptive and exteroceptive) surfaces (Barbas, 2015; Beul and Hilgetag, 2015; Katsumi et al., 2021). Agranular regions are characterized by poor differentiation between laminar layers, the lack of a layer IV, and are relatively further away from sensory surfaces (Beul and Hilgetag, 2015). It is proposed that information coming into the brain via the sensory surfaces (e.g., glucose concentration) is first passed through proximally located granular layers, and summaries of that information occur as it passes through to more agranular regions of the brain. Interoceptive predictions (e.g., low blood sugar/hunger) originate from the deep layers of those agranular brain regions, such as the anterior insula and anterior cingulate cortex (Barrett and Simmons, 2015). Under this framework, actions (e. g., release glycogen or find food to consume) ultimately result from abstract predictions that originate in agranular brain regions and are relayed to peripheral targets (i.e., internal organs and muscles) via subcortical structures such as amygdala and primary motor cortex. Afferent interoceptive information can reach the brainstem via several pathways, and is initially relayed via the subcortical nuclei, including the nucleus of the tractus solitarius (NTS), parabrachial nucleus (PBN), and periaqueductal gray (PAG). Interoceptive prediction errors reach the cerebral cortex in the granular dorsal posterior insula (dpIns; Kleckner et al., 2017). Predictions and prediction errors are not compared once, but instead are iteratively compared across regions as signals travel from the most granular structured regions to the most agranular structured regions. Importantly, the comparison of prediction and afferent signals may occur even before information reaches the brain (Shaffer et al., 2023), therefore nearly all afferent interoceptive information can be considered *interoceptive prediction errors*.

Critically, when predictions and prediction errors are compared, they are not all weighted equally. Instead, the brain can amplify and attenuate signals to prioritize relevant or important information over irrelevant or less important information, a process known as precision weighting (Clark, 2017). For example, signals may be amplified due to salience, novelty, or survival value, amongst other previously learned contextual factors (Pezzulo, 2014; Pezzulo et al., 2018). This weighting or amplification can result in a more precise signal, which can be conceptualized as a reduction in variance in that signal. Additionally, afferent signals (i.e., prediction errors) have precision (i.e., inverse variance) that can be dictated by the source of the signals. Some signals may have a high signal-to-noise ratio, making them inherently precise. For instance, when our heart is beating quickly, the “signal” of a heartbeat is more easily distinguished from “noise” (e.g., other bodily sensations) because of increased contractibility of the heart muscle. In other words, the cardiac signals are likely much more precise when beating fast, relative to at rest, when cardiac signals might be less precise. Importantly, an interoceptive prediction error with greater precision will have a greater impact on future interoceptive predictions because it allows for better updating of the interoceptive model (Ainley et al., 2016). Alternatively, if an afferent interoceptive signal is noisy, but also relevant to well-being, precision weight interneurons can alter the post-synaptic gain of the prediction error signal, amplifying the signal (i.e., increasing precision) and therefore increasing its impact when updating predictions. In either case, prediction error signals with greater precision will have more influence on the updating of priors (i.e., altering future predictions more), and therefore allow for better updating of the interoceptive model of the body (i.e., changes in inference). This allows the brain to better select the needed visceromotor control to adjust the physiological variable of interest (i.e., changes in action) back toward the desired allostatic state.

Consider the visual and skeleto-motor systems as proxies for the interoceptive and visceromotor system, respectively. You are given the task of navigating from one side of a room to the other without hitting any furniture. In a very well-lit room, you would be certain of where the furniture was located (strong inference) and know exactly where to step (efficient action). You could walk from one side of the room to the other with no missteps – expending only the energy needed to perform the task at hand. When the room is completely dark your visual system (interoception) will provide no information about the locations of furniture (low interoceptive precision), you cannot predict where the furniture is (poor inference) and you are thus unsure where to step (inefficient action). It would likely take you a long time to navigate across the room, and you would do so inefficiently by making several missteps. You could perhaps rely on your experience of typical room layouts and furniture placements to better inform your actions (priors can inform inference), increasing efficiency of navigation. If you did not have priors to inform your predictions, and thus actions, you could repeatedly walk through the room in darkness and learn to navigate it (i. e., improve the precision of your priors). In that case, you would begin by making several missteps (prediction error) which you could use to gradually update your model of the layout of the room (learning). Some of your missteps will be more informative than others; for instance, you may bump into a small piece of furniture with clear boundaries (precise information) or you may graze a piece of furniture with no clear indication of how big it is and where its boundaries lie (imprecise information). In this situation you would more heavily weight the more precise information when making the next prediction of where to step (precision weighting). In all cases where we provide better updates for inference and information to select actions, greater precision will lead to reduced prediction error in the future, and better allostatic efficiency.

### 2.2. Dimensions of interoception

While interoception refers to a broad process by which the brain perceives, interprets, and models the internal state of the body, it has typically been studied by decomposing it into several distinct dimensions (Garfinkel et al., 2015a; Pollatos and Herbert, 2018; Suksasilp and Garfinkel, 2022). For the purposes of this review, we will focus on three primary dimensions of interoception: sensibility, accuracy, and meta-awareness.

Interoceptive *sensibility* is the self-perceived ability to focus on or be cognizant of internal bodily sensations (Critchley and Garfinkel, 2017; Garfinkel et al., 2015b; Suksasilp and Garfinkel, 2022). This is a subjective measure of interoception which relies solely on self-report. Common instruments used to measure interoceptive sensibility are the Multidimensional Assessment of Interoceptive Awareness (MAIA-2; Mehling et al., 2018), Interoceptive Accuracy Scale (IAS; (Murphy et al., 2020), and Body Perception Questionnaire (BPQ; Porges, 1993). Together these instruments ask participants to rate themselves in areas such as how much attention they pay to internal sensations, how they interpret those sensations, how much they use the sensations to inform actions, and how much they trust them.

Interoceptive *accuracy* is an objective measure of interoception and refers to the ability to accurately detect internal bodily sensations (Garfinkel et al., 2015b). Some of the most common paradigms for measuring interoceptive accuracy include the Heartbeat Detection Task (Whitehead et al., 1977), Heartbeat Counting Task (Schandry et al., 1993), and Heartbeat Tracking Task (Legrand et al., 2022). Each of these tasks have strengths and weaknesses both in their ease of implementation and the quality of the measurements (for discussion see Brener and Ring, 2016). In the Heartbeat Detection task, participants report when exteroceptive cues are presented in-sync or out-of-sync from their own heartbeat. Alternatively, in the Heartbeat Counting Task, participants attempt to count the number of their own heartbeats in each window of time, which is then compared to the actual number of beats. In the Heartbeat Tracking Task, participants focus on their heartbeat and then are presented with a set of auditory cues that they must determine is faster or slower than their own heartrate. Thus, the Heartbeat Detection Task appears to be the least susceptible to interference based on prior knowledge of one’s resting heart rate, while being more difficult to implement compared to the Heartbeat Counting or Heartbeat Tracking tasks. While the historical focus in interoception accuracy has been on the interoception of the heart, there have also been recent attempts to examine both respiratory (Nikolova et al., 2022) and gastric interoception (Mayeli et al., 2023). An additional novel measure of interoceptive accuracy that could be particularly relevant to pregnant women is the detection of fetal movements and uterine contractions.

Interoceptive *meta-awareness* (sometimes *interoceptive insight*) is the metacognitive awareness of interoceptive accuracy (i.e., self-reported confidence in accuracy of interoception judgements; Garfinkel et al., 2015b). Typically, interoceptive meta-awareness can be collected simultaneously with interoceptive accuracy tasks by probing participant’s confidence in their interoceptive judgements and then assessing the relationship between those confidence ratings and participant’s accuracy. Higher meta-awareness would be reflected in positive correlations between participant’s confidence in their judgements and the accuracy of their judgements.

Measures of sensibility, accuracy, and meta-awareness have been shown to generally correlate, but only in people with high levels of interoceptive accuracy (Garfinkel et al., 2015b). Without considering level of interoceptive accuracy, there may be weak positive associations between interoceptive accuracy and sensibility (Calì et al., 2015), but it has been suggested that methods require further refinement to accurately classify individual differences in interoception (Murphy et al., 2019). Of the three dimensions, we would suggest that interoceptive accuracy most closely assesses the precision of interoceptive modeling. Importantly, there is some evidence that interoceptive accuracy may be consistent across different bodily systems (e.g., cardiovascular and gastric; Herbert et al., 2012). Therefore, the broader construct of interoception, that we reference in this manuscript, is the underlying latent process, only some of which we may be consciously aware, and is not equivalent to any one of the dimensions alone.

## 3. Conceptual framework for interoception and peripartum depression

Pregnancy and new motherhood are periods of dynamic and significant physiologic change that generate a metabolic strain on the mother (Kazma et al., 2020; Mockridge and Maclennan, 2019). These changes occur across nearly every physiological system, including the cardiovascular system (Carlin and Alfirevic, 2008; Soma-Pillay et al., 2016), gastrointestinal system (Cowardin et al., 2023) and glucose regulation (Lain and Catalano, 2007), and are reflected in an increase in both basal metabolic rate and total energy expenditure (Berggren et al., 2015; Forsum and Löf, 2007). This metabolic strain could be buffered by improvements in interoception during pregnancy and the postpartum to protect against depression risk or could lead to allostatic dysregulation and increased depression risk if the improvements are not sufficient.

**Fig. 2.**
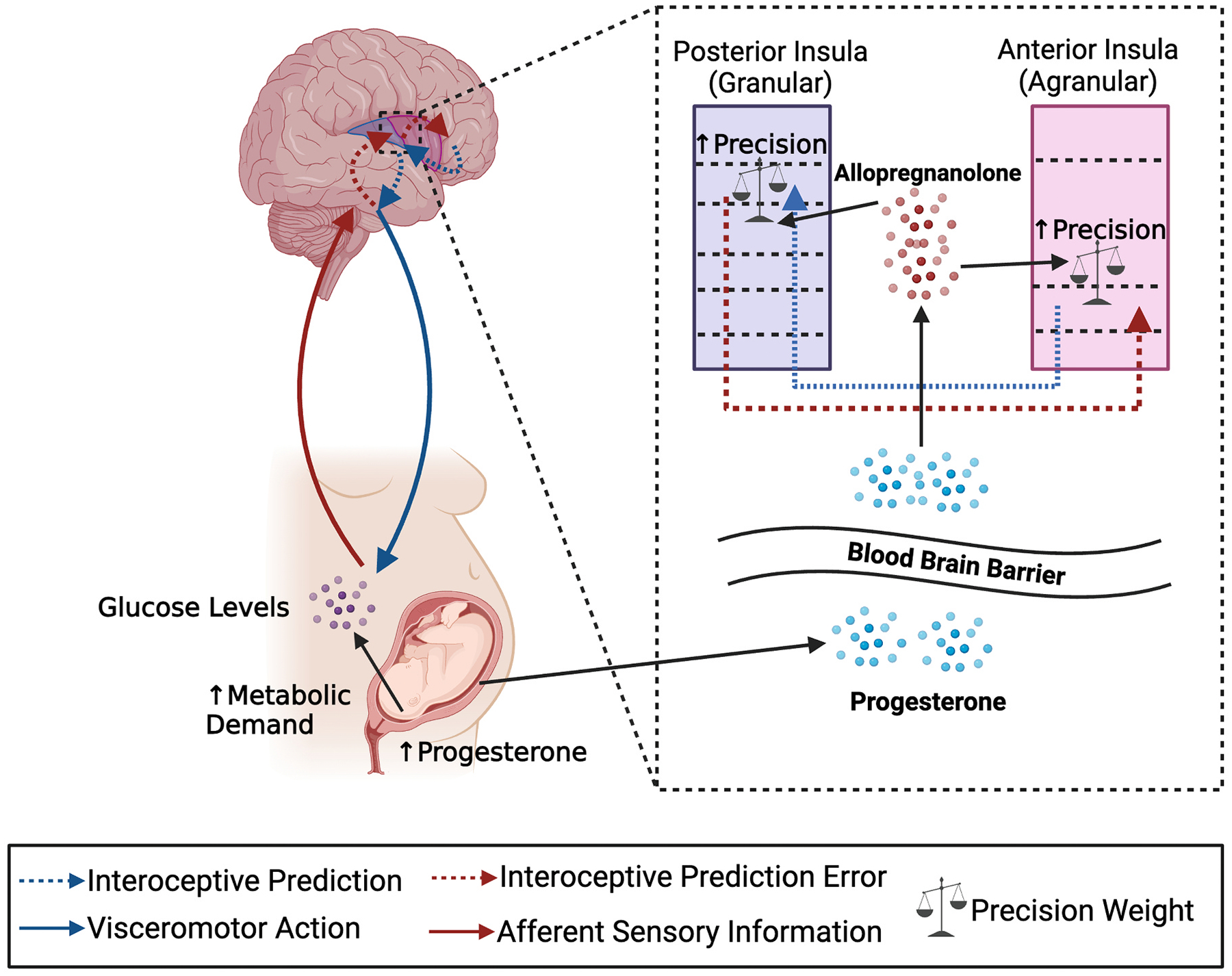
Allostasis and interoception during pregnancy function through predictive processing. The computations underlying predictive processing are facilitated by the cyto-architectural organization of the brain. Interoceptive prediction signals (blue dashed arrows) originate in the most agranular regions and are relayed to regions with increasingly granular structure, where they are continually compared with afferent sensory information and prediction errors (red arrows). In the periphery, these predictions drive bodily actions (blue solid arrow). For example, interoceptive predictions may be generated in the deep layers of the anterior insula which has an agranular (i.e., less distinct layer organization) structure (shown in pink). These predictions travel to the superficial layers of the posterior insula which has a more granular laminar structure (i.e., six distinct and well-organized layers of neurons; shown in purple). Here the prediction meets interoceptive information coming from the body (i.e., interoceptive afferents – solid red arrows). The afferent signals and prediction signals are compared, and the difference is computed as a *prediction error* (red dashed arrows). Prediction errors are then sent from superficial granular layers in the posterior insula to deep agranular layers in the anterior insula. Interneurons (i.e., precision weights) within these regions can alter the precision of prediction and prediction error signals. The precision weight interneurons play a crucial role in balancing the impact of predictions and prediction errors in updating future predictions. We suggest that during pregnancy, increased levels of progesterone cross the blood brain barrier to create increased levels of allopregnanolone in the brain. The effects of allopregnanolone on GABAergic interneurons has the potential to increase the precision weight of interoceptive prediction errors. Prediction errors with greater precision will update future predictions more efficiently and effectively.

The potential for plasticity of interoception during pregnancy (Murphy et al., 2017) may be enabled by a suite of neurobiological and hormonal changes (Carmona et al., 2019; Servin-Barthet et al., 2023). Specifically, we suggest that changes in brain structure and function that are characteristic of pregnancy, as well as changing concentrations of hormones, lay the foundation to support better interoceptive functioning necessary during this stage of life (Fig. 2). If so, heightened interoception would provide the brain’s internal model with more precise information about the current state of the body (Ainley et al., 2016; Barrett and Simmons, 2015). This greater precision would, in turn, allow the brain to anticipate and attempt to meet the impending needs of the body more efficiently (i.e., efficient allostasis). As previously reviewed, by meeting the needs of the body more efficiently, there would be a decreased risk for depressive symptoms. Alternatively, when interoception fails to accurately model the body, or the internal model becomes insensitive to afferent interoceptive information, there is increased risk for depression. As such, focusing on interoceptive mechanisms during pregnancy and the postpartum can provide novel insight into the mechanisms underlying both protective and risk factors for peripartum depression, while also informing potential novel prevention and treatment approaches.

We can return to our previous simplified example of glucose regulation, to map out the conceptual framework more concretely. Note that while this conceptual framework does apply to both pregnancy and postpartum, for the purposes of illustration and because more is known about interoception during pregnancy than in the postpartum, the remainder of this section and the next sections that follow (Sections 4–6) will focus on pregnancy, before we specifically describe how the framework is likely to operate in the postpartum (Section 7). During pregnancy, amongst the many changes a mother will experience, one is an increase in demand for glucose, evidenced by decreases in fasting glucose levels over the course of pregnancy (Hadden and McLaughlin, 2009; Riskin-Mashiah et al., 2011). The change not only introduces a physiological challenge, but also new bodily conditions that differ from previous learned metabolic states (i.e., increased uncertainty/reduced precision). Failing to update predictions to drive adaptive insulin levels may lead the mother to sustain glucose levels that are insufficient to support both her and the developing fetus. The consistent low levels of glucose would likely cause sustained prediction error, which may be experienced as negatively valanced affect (see Section 2). Moreover, the failure to generate actions to modulate glucose levels or metabolism (e. g., eating or changing insulin levels) may lead to sustained feelings of fatigue – another core feature of depression. Indeed, a recent meta-analysis found gestational diabetes, an inability to regulate glucose during pregnancy, is positively associated with depressive symptoms (OuYang et al., 2021).

**Fig. 3.**
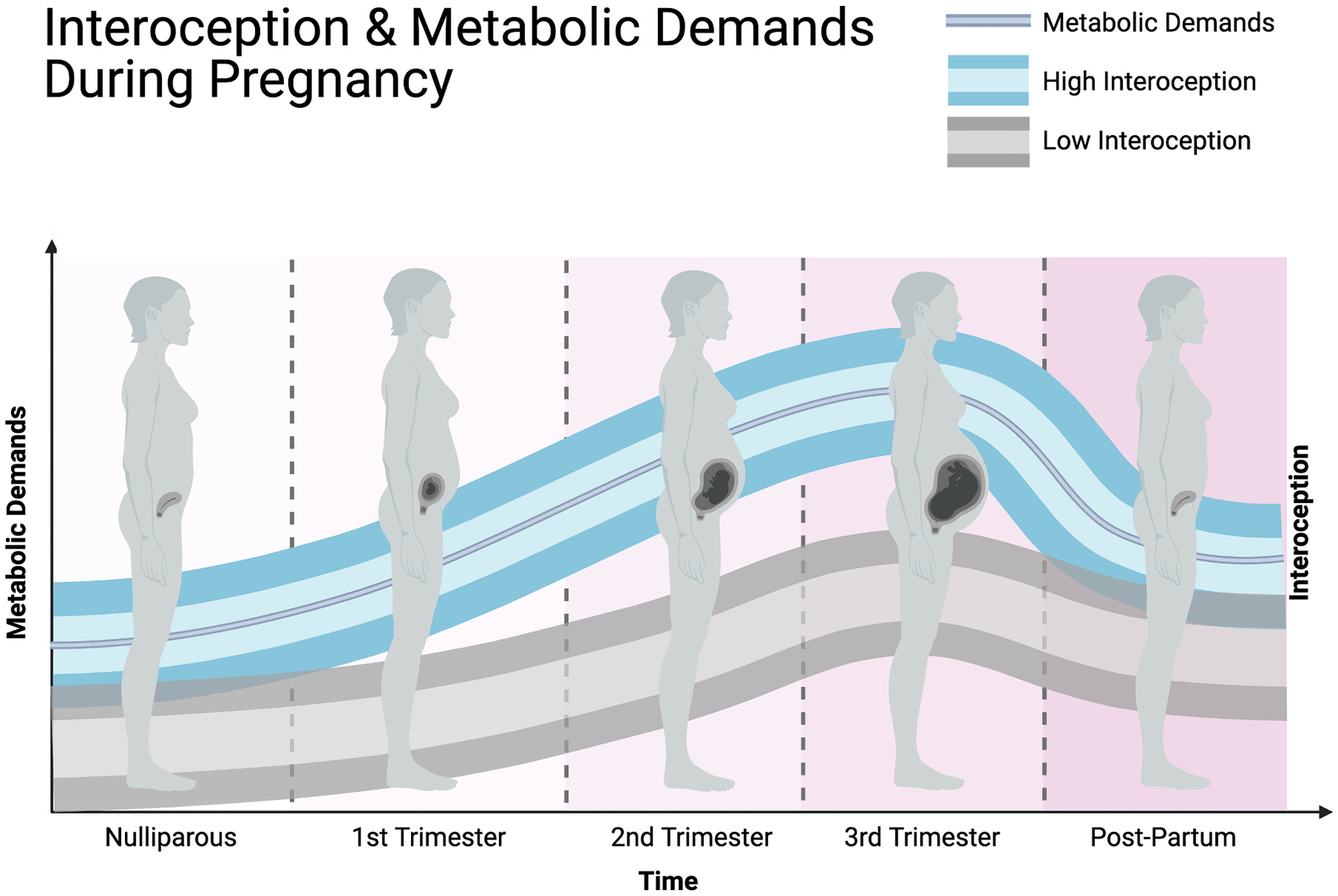
The average metabolic demands (dark blue line) increase during pregnancy. Based on theoretical models and empirical evidence, we suggest that greater discrepancy between interoception and metabolic demands confers greater risk for depressive symptoms and peripartum depression. Prior to pregnancy, individual differences in interoception exist, such that some women will have higher interoception (blue bordered range), than other with lower interoception (gray bordered range). Unpredictable environments (e.g., early life adversity; ELA) may be one key factor driving lower interoception. We suggest that there is typically a increase in interoception across gestation which attempts to match the increasing metabolic demands of pregnancy. Enhancements in interoception, in turn, help to prevent depressive symptoms by providing more precise modeling of the body to enable persistent allostatic efficacy. During pregnancy, we suggest these differences between the women with high and low interoception become amplified because women with lower interoception (e.g., ELA exposed women) will have difficulties enhancing interoception to keep up with the metabolic demands of pregnancy. This difficulty is proposed to stem from ELA related unpredictable environments leading to weak priors, and to certain biochemical mechanisms that are suppressed following ELA. For simplicity, the ranges representing interoception are meant to illustrate the mean level of each condition, but there is meaningful variance in interoception during baseline and pregnancy, as well as in the rate of increase across gestation.

We hypothesize that the neurobiological changes induced by pregnancy serve to facilitate improvements in interoception, such that pregnant women’s brains are particularly well equipped to deal with changing interoceptive information and regulatory needs of the body. Enhanced interoception would better place pregnant women to 1) update their predictions (i.e., anticipating increased need for glucose over the next 9+ months), reducing prediction error, and 2) increase their sensitivity to interoceptive information enabling faster and more adaptive allostatic actions to remediate prediction errors. Increased precision weighting of interoceptive prediction errors may serve as a mechanism to address the increased uncertainty in interoceptive afference during pregnancy. In this way, under optimal conditions, enhanced interoception during pregnancy can address the increased metabolic demands of pregnancy, helping to buffer against allostatic inefficiency, which may underlie some of the core features of peripartum depression (Fig. 3). In the following sections, we will outline how the neurobiological and hormonal changes related to pregnancy can support enhanced interoception, before then showing in subsequent sections how life experiences that increase uncertainty (e.g., early life adversity) may lessen interoceptive adaptations during pregnancy, increasing peripartum depression risk.

## 4. Pregnancy-related changes that support interoceptive plasticity

### 4.1. Neural changes

Despite the lack of known risks to mother or fetus, there has been limited structural and functional magnetic resonance imaging (MRI) data collected in pregnant women (Newmark et al., 2019). As a result, much of the MRI literature has examined women in the postpartum period or has compared brain scans collected pre- vs. post-pregnancy, with only a few studies having scanned women during any trimester of pregnancy. These limitations notwithstanding, this literature to date has highlighted neural changes associated with pregnancy that are both relevant and informative for interoception.

#### Structural brain changes across pregnancy.

One of the first studies to image the brain during pregnancy and again postpartum reported that pregnancy was associated with a lower total brain volume that was lowest at term and was completely reversed at 6 months postpartum (Oatridge et al., 2002). Since this initial paper was published, there have been approximately six sets of published longitudinal analyses examining brain changes pre- to post-pregnancy (Carmona et al., 2019; Hoekzema et al., 2017, 2020, 2022; Martínez-García et al., 2021; Spalek et al., 2024). The findings from Oatridge et al. were generally replicated and expanded upon to show pregnancy-related reductions in gray matter across a wide range of brain regions including the medial frontal cortex, lateral prefrontal cortex, anterior cingulate cortex (ACC), posterior cingulate cortex (PCC), precuneus, temporal cortex, and insula (Carmona et al., 2019; Hoekzema et al., 2017, 2020, 2022). Importantly, nearly all the regions that have structural decreases associated with pregnancy, such as the insula, anterior cingulate cortex (ACC), and anterior midcingulate cortex (aMCC), also play prominent roles in interoceptive processing. Structural reorganization in these brain regions during pregnancy is widely thought to reflect neuronal specialization (Pawluski et al., 2022) and such changes have been associated with enhancements in a variety of behaviors that are of ecological significance to a new mother (e.g., postpartum maternal attachment to the baby; Hoekzema et al., 2017). While the functional significance of these structural changes is largely unknown, reviews of this literature generally posit that the changes are likely to be adaptive for the mother (e.g., adopting new maternal behaviors; Hoekzema et al., 2017; Luders et al., 2022; Pawluski et al., 2022), although others have also suggested that at least some of the structural brain changes may pose risks for maternal mental health (Cárdenas et al., 2020). While no studies have examined links between structural changes and interoception during pregnancy, we hypothesize that they would be positively correlated.

At least one study (Hoekzema et al., 2017) found a reversal of gray matter changes in the postpartum period that were originally reported by Oatridge et al., while others have suggested that some of the pregnancy-related reductions in gray matter persist even 6 years postpartum (Martínez-García et al., 2021). This suggests that at least some of the pregnancy-related brain changes may not be completely reversible. Additionally, decreases in volume may not be ubiquitous throughout the brain, as the pituitary gland has been shown to have greater volume in pregnant than non-pregnant women in a cross-sectional analysis, but this was driven by the anterior portion, while the posterior pituitary follows the more widely seen pattern of pregnancy-associated volume reduction (Benson et al., 2023). Such results highlight the need to understand the region- specific effects of pregnancy on the brain. The findings on this body of literature are further limited by the fact that no published study to date has examined trimester specific effects on brain structure (Puri et al., 2023).

#### Functional brain changes across pregnancy.

The findings suggesting structural neural adaptations to pregnancy are paralleled by a small body of literature documenting functional changes to the brain during pregnancy or in the early postpartum period. In one relevant study, functional (f)MRI scans collected pre-conception and again postpartum showed evidence for increased within-network functional connectivity (i.e., increased network coherence) within the default mode network (DMN; Hoekzema et al., 2022). Increased network coherence within the DMN is relevant to interoceptive processing, as the DMN is a critical component of the interoceptive-allostatic brain network (Kleckner et al., 2017; Zhang et al., 2023).

In another study, electroencephalograms (EEG) collected during pregnancy revealed evidence for region specific changes in brain activity (increased as well as decreased) at rest, relative to non-pregnant women (Luo et al., 2020). Two additional EEG studies have provided evidence for altered amplitudes of event related potentials (ERPs) in pregnant vs. non-pregnant women (Raz, 2014) and in first-time pregnancy vs. multiparious women (Rutherford et al., 2019). In the first of those studies, women in their first or second trimester, relative to women who were not pregnant, were found to have a reduced P300 amplitude in response to affective, but not non-affective, stimuli (Raz, 2014). Given the association of the P300 with attentional processes (Gray et al., 2004; Patel and Azzam, 2005; Polich, 2007), this study suggests that pregnancy may reduce or at least alter attentional processes for affectively valanced information from the environment (also known as exteroceptive information). In the second of those studies, the P300 ERP component was found to be higher in amplitude in first-time pregnant women relative to multiparous women, for both social and non-social stimuli (Rutherford et al., 2019). These results suggest that pregnancy does impact attentional processes indexed by the P300 ERP, but the direction of those effects may be variable across studies, possibly due to differing stimuli used.

More recent EEG research revealed that pregnancy was associated with lower interhemispheric coherence between frontopolar areas, and higher coherence between frontopolar and parietal areas, as compared to the coherence in these regions at 3 months postpartum (Sandoval et al., 2023). Coherent activity between cortical regions is involved in synchronizing and combining information processed in different brain regions into unified concepts or perceptive experiences. Critically, frontoparietal regions are also involved in the generation of multimodal predictions used in interoception (Barrett and Simmons, 2015). As these regions are critical for multimodal integration and representation, these findings bring up the intriguing possibility that pregnancy is associated with changes in basic sensory processing, including interoception.

The data on functional neural changes during pregnancy may be limited, but the existing literature broadly supports the hypothesis that such changes could act to buttress interoception during pregnancy. While much of the literature assessing behaviors linked to maternal brain changes have focused specifically on attachment and caregiving, we suggest that a greater focus on how brain changes are linked to basic sensory (including interoceptive) processing in expecting mothers is warranted.

#### Pregnancy as a sensitive period of neural development.

Another interesting feature of the nascent literature on pregnancy-associated neural changes is how closely they parallel those occurring at other sensitive developmental stages, particularly adolescence. Specifically, decreasing cortical thickness accompanied by broad increases in functional connectivity match the neural changes which characterize adolescence (Carmona et al., 2019; Pawluski et al., 2022), a stage of development widely recognized as a sensitive period for memory, reward learning, cognition, and social sensitivity (Blakemore and Mills, 2014; Fuhrmann et al., 2015; Larsen and Luna, 2018). When structural and functional neural reorganization is observed during adolescence, it is widely viewed as a developmental adaptation to support behaviors which are changing rapidly at that time (Callaghan et al., 2022). We suggest that such neural changes, when occurring during pregnancy, similarly represent a sensitive period of development, in which the mother is building a behavioral and neurobiological repertoire to fulfill the demands of pregnancy and parenting. We propose that one of the core maternal behaviors improved by these widespread neural changes is interoception.

### 4.2. Potential endocrine pathways affecting interoception

Pregnancy is charactered by massive fluctuations in circulating hormones, unparalleled at any other stage of life. These hormones include cortisol, corticotrophin releasing hormone, progesterone, estrogen, and human chorionic gonadotropin hormone (hCG; Schock et al., 2016), which can have a wide range of effects both centrally and peripherally in both the mother and fetus, and which may underlie interoceptive changes in the mother that occur throughout pregnancy. The following section will focus on progesterone and estrogens and their links to interoception.

#### Progesterone.

Progesterone steadily increases during pregnancy, particularly during the second and third trimesters, before peaking and then rapidly decreasing postpartum (Schiller et al., 2015; Ziai et al., 1994). The majority of progesterone during later pregnancy is produced by the placenta (Tuckey, 2005). Regardless of where it is produced, progesterone’s small size and lipid solubility enable its easy free transport across the blood brain barrier (BBB) where it can then have central effects (Guennoun, 2020). A key neuro-steroid derived from progesterone is allopregnanolone, a positive allosteric modulator (Brunton and Russell, 2010). Allopregnanolone binds to GABA_A_ receptors in the brain, increasing agonist affinity and/or efficacy (Gago et al., 2004; Majewska and Vaupel, 1991; Paul et al., 2020; Putnam et al., 1991). GABA functions as one of the primary inhibitory neurotransmitters in the brain and this GABAergic inhibition is involved in both the opening and closure of sensitive periods across the sensory cortex (Hensch and Bilimoria, 2012; King et al., 2014). While non-human animal levels of cortical GABA do not change during pregnancy (Smolen et al., 1993), human serum and central allopregnanolone levels increase throughout pregnancy (Concas et al., 1998; Luisi et al., 2000). Accordingly, there is increased potential for inhibitory GABAergic signaling in the brain during pregnancy due to the allopregnanolone-induced improvements in the efficacy of the GABAergic receptors.

Cortical GABAergic neurons are thought to be important for interoceptive functioning. Evidence from rodent models has revealed a critical role for GABA in multimodal integration of signals within the insula (Gogolla et al., 2014). Such signal integration, in turn, underlies accurate interoceptive modelling across multiple sources of interoceptive information. In humans, a simultaneous fMRI-MRS study found that GABA concentrations within the insula were positively associated with functional activity of the insula during interoceptive processing (Wiebking et al., 2014). In that study, both GABA levels and functional activation of the insula were positively associated with interoceptive accuracy (heartbeat counting), suggesting an important role for inhibitory neuronal signaling within the insula for interoception. We propose that increased concentrations of allopregnanolone within the insula, driven by surging progesterone during pregnancy, could have similar beneficial effects on interoceptive processing in pregnant individuals. Indeed, serum allopregnanolone levels in non-pregnant humans have been associated with altered insula and amygdala activation during an emotion appraisal task (Sripada et al., 2013), suggesting that allopregnanolone has the potential to influence the function of brain regions implicated in interoceptive functions in humans.

Mechanistically, the relationship between increased inhibitory signaling and improved interoception may be realized via changes in the precision (i.e., inverse variance) of interoceptive signals. Pyramidal interneurons play a key role in the precision weighting of interoceptive signaling (Barrett and Simmons, 2015), and these interneurons appear to rely on GABAergic signaling to modulate post-synaptic gain (i.e., amplification or attenuation) of signals (Quattrocki and Friston, 2014). Indeed, reduced inhibition can lead to imprecise signaling or greater “noise” in neural circuits (Nelson and Valakh, 2015). Increased precision can amplify relevant interoceptive signals and attenuate irrelevant interoceptive signals (i.e., improved signal-to-noise ratio; Owens et al., 2018). By improving the signal-to-noise ratio, the brain is better equipped to predict physiological needs and prepare to meet those needs before they arise, resulting in greater metabolic efficiency. Thus, allopregnanolone modulation of GABAergic neurons may directly affect the precision weighting of interoceptive signals, and by extension the efficacy of interoceptive processing during pregnancy.

More broadly, the effects of allopregnanolone on interoceptive processing may also be realized via alterations to excitatory-inhibitory (E/I) balance of neural circuits throughout the brain. E/I balance refers to the combination of inhibitory and excitatory neural firing which maintain effective functioning by preventing over-inhibition and run-away over-excitation (Sukenik et al., 2021). From an active inference view, E/I balance is likely related to the precision of afferent prediction errors (Kondo et al., 2018). The underlying computational processing that underlies active inference relies on a combination of excitatory signaling and inhibition of confirmed prediction signals (i.e., accurately predicted predictions not being fed-forward). As such, E/I balance can be thought to represent more efficient neural predictive processing. In humans, gamma power is a commonly used metric of E/I balance for neural circuits (Mueller-Buehl et al., 2023; Reh et al., 2020), and is positively associated with a number of GABA receptors in the brain (Kujala et al., 2015). Importantly, gamma power in humans during interoceptive tasks is associated with interoceptive accuracy scores (Fukushima et al., 2019), and animal models demonstrate that allopregnanolone levels can directly impact gamma oscillations in the brain (Ferando and Mody, 2013). Therefore, increased levels of allopregnanolone during pregnancy are a potential pathway by which progesterone surges could indirectly affect the E/I balance of neural circuits leading to greater neuronal precision and better interoception.

#### Estrogens.

Human pregnancy is also characterized by dramatic increases in estrogens, which peak at the end of the third trimester (Tal and Taylor, 2000). During pregnancy, estradiol (E2) is thought to stimulate the neural serotonergic system and the placenta to synthesize serotonin (Hudon Thibeault et al., 2019). Serotonin has been implicated in both interoceptive processing and autonomic regulation (Berntson and Khalsa, 2021; Watts et al., 2012; Yabut et al., 2019). For example, acute increases in serotonin levels have been shown to improve interoceptive meta-awareness; Livermore et al., (2022). In addition to interoceptive effects, serotonin may also directly impact allostatic regulation as it can regulate appetite and energy expenditure through the central nervous system and peripherally regulate adipose tissue (Yabut et al., 2019). Together these data suggest a critical role for serotonin in both interoceptive and allostatic functioning.

Increased levels of serotonin from the placenta and estrogen-stimulation within the CNS could be another pathway by which hormones help to enhance interoception and maintain allostatic efficiency during pregnancy. The combined endocrine effects during pregnancy have the potential to increase interoceptive precision, which would support allostatic efficiency in the context of increased metabolic demand – ultimately buffering against the risk for prenatal depressive symptoms.

## 5. The effects of early life environments on interoception during pregnancy and links to peripartum depression

The framework we propose in this review can also be used to understand why certain early life environments, e.g., early adversity exposure, increase risk for peripartum depression. It has been documented that women who were exposed to early life adversity (ELA) are at greater risk for peripartum depression (Tebeka et al., 2021; Wajid et al., 2020). While many factors may influence the risk for peripartum depression, ELA is of particular significance because it can represent transgenerational transmission of adversity (Sawyer et al., 2019). In general, individuals exposed to adversity are reported to have worse interoception as adults (outside of pregnancy; Bonaz et al., 2021; Schaan et al., 2019). In order to understand how ELA exposure could increase risk for peripartum depression via changes to interoception, it is worth focusing on a specific feature shared across many forms of adversity - an unpredictable environment (Davis and Glynn, 2024).

Our ability to form interoceptive predictions and enact motor control is learned and refined throughout development, and as such, early-life experiences play a particularly important role in determining individual variability in interoceptive processing (Atzil et al., 2018). At birth, we are unable to fully regulate our own internal milieu (Hofer, 1994), and therefore must rely on caregivers to help regulate our allostasis (Atzil et al., 2018). These inputs we receive during early childhood serve as the initial priors for interoceptive and allostatic predictions later in life. An optimal caregiver and environment have high sensitivity and consistency to meet our metabolic needs, and as such “train” our interoceptive and allostatic model to be sensitive and accurate (i.e., high precision). However, unpredictable environments (e.g., inconsistent caregiving, food scarcity, political turmoil) introduce additional variability (i.e., low precision) in learning to predict our interoceptive state. As discussed earlier, imprecise interoceptive predictions lead to inefficient allostatic regulation. Indeed, exposure to childhood adversity generally has been associated with impaired interoception in adults (Bonaz et al., 2021; Schaan et al., 2019), which in turn is associated with depression (Avery et al., 2014; Brand et al., 2023; Dunne et al., 2021; Eggart et al., 2019).

Returning to our example of walking through a dark room, consider walking through the same dark room every single day. Eventually, you would incorporate prediction errors and past experience to form an accurate internal model of the room and to proficiently navigate the room. Now let us consider an example wherein each night someone rearranged the room. Each time you return, the room is different than when you last encountered it. In this situation you would no longer proficiently navigate the room, although you may learn some strategies to attempt to minimize prediction errors (e.g., walking slowly). This describes the effect of environmental unpredictability on the internal model used to maintain interoception and allostasis. We suggest environmental unpredictability introduces high levels of uncertainty, which interferes with interoceptive modeling and maximizing allostatic efficiency. We propose that this occurs because uncertainty in the environment will be encoded as low-precision priors for future predictions, which are more likely to lead to prediction errors. Of course, there will be heterogeneity in outcomes, as some strategies may overcome low-precision priors, but often when someone with low-precision priors enters a higher fidelity environment (i.e., predictable situations), these priors become maladaptive, as they no longer match the features of the environment.

**Fig. 4.**
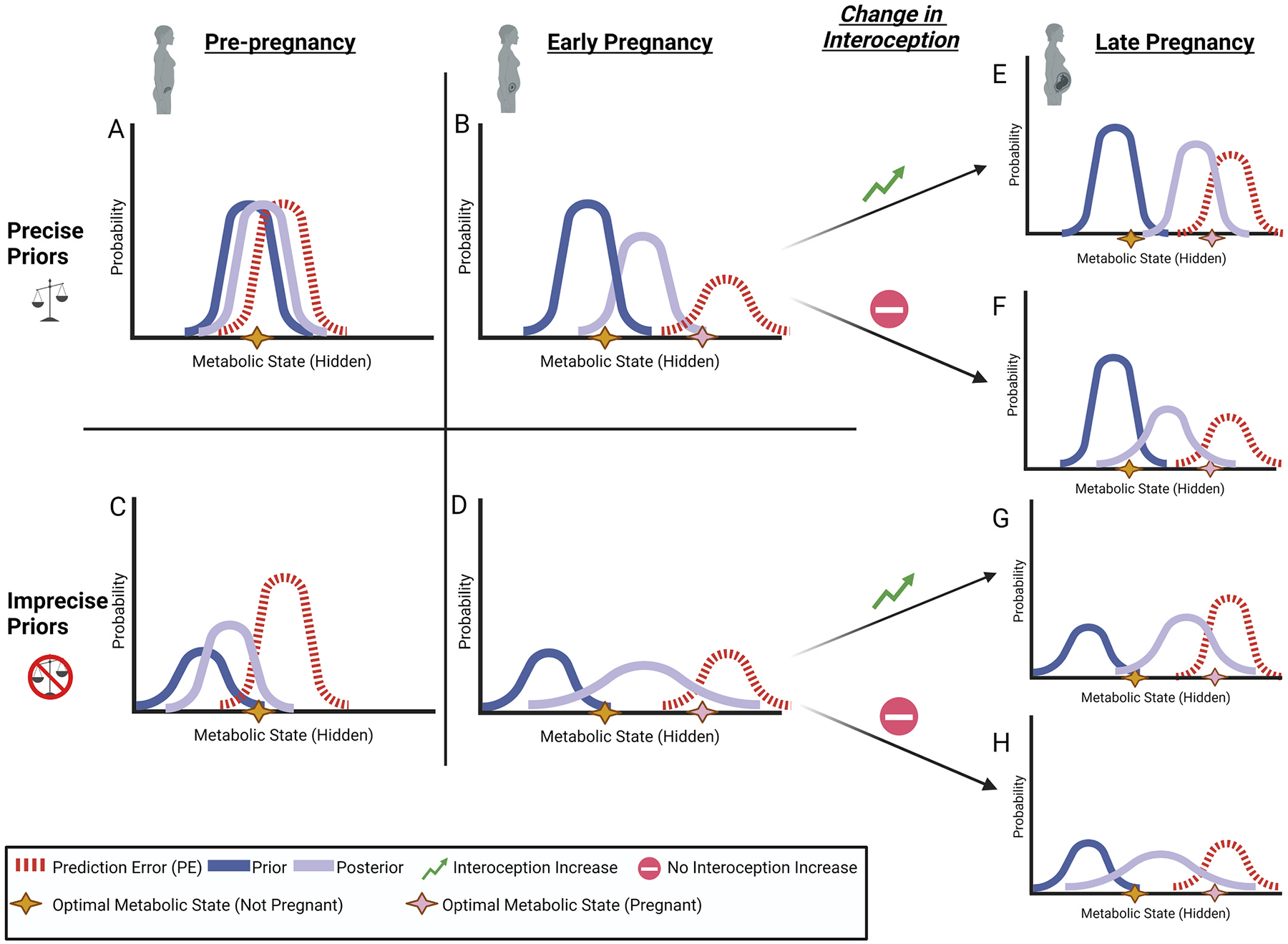
A combination of differences in the strength of interoceptive priors and changes in interoceptive precision during pregnancy may result in individual differences in depressive symptoms during pregnancy. The brain uses Bayesian estimation to infer the current hidden (i.e., not directly observable) state of the of the body using prior knowledge to make predictions (i.e., prior distribution; solid dark blue line) and compares that to the current prediction error (i.e., unpredicted deviations in afferent interoceptive signals; dashed red line) to generate an inference (i.e., posterior distribution; solid lavender line). For any given context there is a theoretical optimal metabolic state for the body to be in (gold star for non-pregnant; pink star for pregnancy). A) In the non-pregnant state, the prior distribution is likely to be centered on a metabolic optimal state, because it serves as an “attractor state” by keeping the body within biological bounds that maintain life. It should be noted that these “optimal states” are completely context dependent (e.g., different for sleep vs. exercise) but for simplicity we depict this as a single point. B) For women with weak interoceptive priors (e.g., growing up in an unpredictable environment), we suggest that the precision of their prior distribution is reduced. This results in a less precise posterior distribution (even when the precision of prediction error is held constant), suggesting a lower likelihood of generating predictions that best maintain the optimal metabolic state. C & D) For all women, the optimal metabolic state of the body shifts to accommodate the additional metabolic needs of pregnancy. Additionally, the novel and variable conditions of pregnancy generate greater, but less precise prediction errors. E & G) As pregnancy progresses, we suggest that the biological mechanisms we reviewed can enhance the precision of interoceptive predictions errors (Arrows 1 & 3), moving the posterior distribution even closer to the optimal metabolic state during pregnancy. F & H) However, for various reason (e.g., ELA exposure, other individual differences) some women may not experience an increase in interoceptive precision (Arrows 2 & 4), which may result in increased risk for depressive symptoms, because their posterior distributions have less precision around the optimal metabolic state, relative to women who experience a improvement in interoception. We suggest this framework of differences in the precision of interoceptive predictions and prediction errors, explains the differences in baseline interoception, as well as a proposed amplification of this difference during pregnancy.

We propose that the increased and variable metabolic demands of pregnancy will further exacerbate the differences between women raised in unpredictable environments and those raised in more predictable environments, increasing their risk for peripartum depression. When a woman becomes pregnant, regardless of her early-life history, there is a drastic change in metabolic demands, which should initially create a sustained increase in interoceptive prediction errors. Importantly, interoceptive priors should be biased to predict biologically optimal states, which may not be true for inefficiently encoded priors. In other words, women raised in predictable environments are more likely to have learned priors for adaptive physiological and metabolic states, while women raised in highly unpredictable environments may have encoded priors which do not generate metabolically efficient states for a given environment. Indeed, research in non-pregnant adult samples has shown uncertainty in childhood to be associated with poor mental and physical health (Glynn et al., 2019; Maner et al., 2023; Spadoni et al., 2022). Weak priors, combined with variable interoceptive afference have a compounding effect on the ability to maintain an efficient metabolic state (Fig. 4).

In addition to the effects ELA-associated unpredictability may have on priors, we can also consider the mechanistic neurobiological impacts of ELA on interoception via interactions with GABAergic systems. For example, rodent models have demonstrated that ELA impairs GABA_A_ receptor expression (Mitchell et al., 2018), which is the same receptor type that allopregnanolone acts upon. As reviewed above, allopregnanolone likely impacts precision weighting of prediction errors to enhance interoceptive signal-to-noise ratios during pregnancy, and changes E/I balance to enhance neuronal precision and interoception. This suggests a potential for women exposed to ELA to benefit less from the effects of increased central allopregnanolone during pregnancy. Following this logic, after ELA, interoception during pregnancy may suffer from both low precision priors (due to unpredictable early environments), and even lower precision prediction errors (due to impaired GABA_A_ receptor expression), relative to pregnant women with low early adversity exposure.

Beyond GABA_A_ receptor expression, GABA levels themselves may be affected by ELA. Evidence in humans suggests that post-traumatic stress disorder (PTSD) is associated with significantly reduced GABA levels specifically within the insula (Rosso et al., 2014), and adults with a history of ELA exposure had lower GABA concentrations within the left superior temporal gyrus (Hepsomali et al., 2023), which sits proximally to the insula. Given the previously discussed association between insular GABA-levels and interoception, this may be another key mechanism by which women exposed to ELA could demonstrate impaired interoception pre-pregnancy, which might become further amplified during pregnancy. Specifically, the combined effects of reduced GABA_A_ receptor expression and lower GABA levels in the insula could have an additive effect, whereby the difference in interoception between ELA-exposed and non-ELA exposed women is not only maintained during pregnancy, but further enhanced.

Ultimately, we suggest that during pregnancy, women with a history of ELA may have low precision in predicting their bodily state, resulting in inefficient allostatic actions and costly prediction error signaling. Given the hypothesized role of metabolic efficiency in depressive symptoms, a pathway of altered interoception during pregnancy would help to explain the elevated rates of peripartum depression that are observed among women exposed to ELA (Tebeka et al., 2021; Wajid et al., 2020).

## 6. Behavioral evidence linking pregnancy to differences in interoception

Although the literature on interoception during pregnancy is sparse and mostly limited to self-report studies of interoceptive sensibility, existing evidence suggests that this domain of interoception may be heightened during pregnancy with some exceptions. In one study (N = 134), women were followed across pregnancy and into the postpartum period (Singh Solorzano et al., 2022). This study found that women reported greater interoceptive sensibility during pregnancy relative to postpartum. Another study (N = 500) compared interoceptive sensibility in pregnant and non-pregnant women and found that pregnant women reported higher scores in the domain of ‘not distracting’, which describes the tendency to not distract from painful or uncomfortable sensations (Crossland et al., 2022). This same group also found that better interoceptive sensibility during pregnancy was related to better antenatal attachment (N = 159; Stafford et al., 2024), and in another large longitudinal cohort (N=253) that better interoceptive sensibility was associated with lower levels of depressive symptoms during pregnancy (Munns and Preston, 2024). An additional study (N = 32) provided evidence that primiparous pregnant women had lower interoceptive sensibility in the domain of ‘attention regulation’, which describes the sustained and controlled attention to body sensations, relative to multiparous pregnant women (Noda et al., 2022). However, as there was no comparison to non-pregnant women, it is unclear what this relative difference with parity says about interoception in pregnancy generally, though one interpretation is that the effects of pregnancy on interoception are cumulative (i.e., increase with parity). That study also used a heartbeat counting task, which is an objective measure of interoceptive accuracy (discussed in Section 2.2; Schandry et al., 1993), but did not find differences in performance on this task across gestational weeks, nor parity, and again, did not make a comparison with a group of non-pregnant women. Critically, many effects of pregnancy on behavioral outcomes are subtle and require large sample sizes to be detected, relative to the more modest sample (n = 32) collected in this study. Furthermore, the heartbeat counting task of interoceptive accuracy can be biased by prior knowledge of one’s resting heart rate, or even average resting heart rates in the population (Brener and Ring, 2016). Given that heartrate increases across gestational week (Loerup et al., 2019), women in later-pregnancy may not perform as well, because they cannot rely as much on previous knowledge about their heartrate, potentially masking effects of improved interoception.

In contrast to the improvements in interoception reported in the general pregnant population, recent emerging research suggests that interoception in pregnancy may be altered in the context of early life adversity exposure, in ways that link to depression. In our recent cross-sectional study (N=192), we observed that pregnancy-related differences in interoceptive sensibility were moderated by exposure to childhood adversity (Savoca et al., 2024). In that study, although there was no main effect of pregnancy on interoception, differences in interoception between pregnant and non-pregnant women were observed at the ends of the adversity spectrum. Specifically, when exposure to childhood adversity was low, pregnant women reported higher interoceptive sensibility in the self-regulation domain than non-pregnant women. In contrast, when exposure to childhood adversity was high, pregnant women showed poorer interoceptive sensibility than non-pregnant women in the attention regulation domain. In turn, lower levels of interoception were associated with greater levels of depressive symptoms. As such, the literature to date provides moderate evidence for an increase in interoceptive sensibility during pregnancy (particularly among individuals with low exposure to early adversity), relative to after pregnancy, or relative to a group of women who are not pregnant; although studies differ in the specific sub-domain of interoceptive sensibility that is enhanced. Moreover, early evidence suggests that exposure to early life adversity may impair interoception in pregnancy. Additionally, concerns about bias in the heartbeat counting task greatly limits our knowledge of how pregnancy affects interoceptive accuracy.

## 7. Interoception and depression in the postpartum period

While our description of the theoretical model has thus far has focused on changes to interoception during pregnancy contributing to perinatal depression risk, the unique neurobiology of the postpartum period is also likely to affect interoception in ways that contribute to postpartum depression risk. In other words, while interoception is likely to contribute importantly to depression risk in both the prenatal and postnatal periods, the precise mechanisms through which changes to interoception contribute to depression risk are likely to differ in the postpartum, relative to what has been described during pregnancy. Indeed, as the rates of depression during the postpartum period are similar to during pregnancy, unique risk and protective factors for depression are likely to be at play during each period (Cheng et al., 2021; Heron et al., 2004; Shorey et al., 2018). In this section we will consider three features of the postpartum state that are likely to impact interoception in ways that could modulate risk for postnatal depression: shifts in hormones hypothesized to support interoception, sustained metabolic demand, changes to the maternal brain.

First, the postpartum phase involves new and rapid changes in physiology (e.g., parturition, hormone shifts, lactation). This rapid change likely produces interoceptive information with increased noise and variability (i.e., low precision). This low precision, as already discussed, presents additional challenges to the new mother to model the metabolic state of her body, rendering it more difficult to maintain allostasis, leaving her vulnerable to depression. For instance, a sudden and rapid drop in progesterone and estrogen post-parturition (Sacher et al., 2020), hormones hypothesized to support enhanced interoception during pregnancy, may lead to relatively reduced interoception postpartum. This could present a time-window in which women struggle to model the state of their body and be at greater risk for depression. As discussed below, this increased risk might be offset by protective features unique to the postpartum period.

Second, while metabolic demands are raised during pregnancy, they remain high throughout the postpartum period, in part, due to lactation (Butte and King, 2005; Dewey, 1997). In fact, lactation may be more energetically costly than any other metabolic state (Dufour and Sauther, 2002; Prentice and Prentice, 1988). These demands will continue to put additional strain on maintaining allostasis, which could increase the risk for depressive symptoms. Despite the metabolic costs of breastfeeding, however, the behavior has been associated with a reduced risk for postpartum depression (Alimi et al., 2022), which may operate through increases in oxytocin (Uvnäs-Moberg et al., 2020; Whitley et al., 2020). Similarly to progesterone and allopregnanolone, oxytocin can modulate GABA_A_ receptors to increase GABAergic functioning (Quattrocki and Friston, 2014), and ultimately protect against postnatal depression. Indeed, increasing oxytocin during breastfeeding and lactation may, in part, compensate for the massive drop in progesterone and allopregnanolone that occurs at parturition, which would otherwise act as a risk factor for postpartum depression. In addition to breastfeeding, social support may also play a key role in offsetting the allostatic strain of the postpartum period. Greater levels of social support have been associated with lower levels of postpartum depression (Vaezi et al., 2019; Xie et al., 2009). Social factors can have a strong impact on our ability to maintain allostasis (Atzil and Barrett, 2017; Theriault et al., 2021). Therefore, if positive social relationships can help a new mother maintain allostatic efficiency, it can help to reduce risk for depressive symptoms, regardless of if there are changes in interoception.

Third and finally, the postpartum period is also characterized by a partial reversal in structural neurological changes in brain regions that were altered during pregnancy (Martínez-García et al., 2021). As reviewed, these include many regions that are critical to interoceptive function. Therefore, as the brain readjusts to a non-pregnant body, with new parental responsibilities, interoceptive circuitry is likely rewiring itself to make adaptive predictions in this new context. Disruptions in the rewiring and remodeling of neural circuitry during this period could impair interoceptive precision, therefore increasing depression risk. However, the association between increases in cortical thickness and interoceptive functioning following pregnancy remains an empirical question to be answered.

Similar to the prenatal period, in the postnatal period we propose that past ELA exposure should also be considered as moderator of risk and protective factors for changes to interoception and ultimately postnatal depression symptoms. As we already reviewed, ELA has been associated with reduced interoception, which we would expect to also be true during the postpartum period due to low precision interoceptive priors acquired throughout life. Additionally, ELA has been associated with reduced basal levels of oxytocin (Ellis et al., 2021). Similarly, to basal reductions of GABA discussed previously, this may be another pathway by which ELA increases risk of depression in the peripartum period, via reduced interoceptive precision. In addition to hormonal disturbances, there is evidence that links ELA to altered metabolism, both generally and specifically in the postpartum period (de Lima et al., 2021). ELA has also been associated with smaller social network sizes in adulthood (Ford et al., 2011), which may limit the potential for social support to help maintain allostasis. Together, this evidence suggests the potential for ELA to impact postpartum depression risk through these pathways, but more research is needed to determine the specificity of these effects on interoception during the postpartum period, and if ELA impacts the partial reversal of neurobiological changes seen in pregnancy.

Finally, it is critical to consider that the risk and protective factors during pregnancy compared to the postpartum period are likely highly individualistic. This means that while some women may suffer from depression during both periods, there are some women who will be depressed during pregnancy and not postpartum, and vice versa. While the multitude of risk and protective factors generate a complex model for risk, the underlying need to maintain precise interoceptive processing to prevent depression remains consistent across all situations.

## 8. Implications for treatment and future directions

If our conceptual framework for proposing that pregnancy-related changes in interoception represent a key mechanism gating the risk for peripartum depression holds true, there are important implications for prevention and/or treatment. In particular, we will highlight the potential roles of behavioral or mindfulness interventions and pharmacological treatments, which are all capable of altering interoceptive functioning (Nord and Garfinkel, 2022; Weng et al., 2021). These domains of treatments may differentially serve an individual’s preferences and circumstances, but we suggest all three necessitate further investigation for potential breakthrough improvements in peripartum depression.

A potentially effective approach for peripartum depression treatment and prevention are behavioral and mindfulness interventions. These treatments may be particularly attractive for women who are hesitant to use antidepressant medications during pregnancy and postpartum (Battle et al., 2013). For example, in non-pregnant women, there is evidence that mindfulness training can improve interoceptive sensibility (Lima-Araujo et al., 2022). Additionally, targeted interoceptive training has been demonstrated to improve interoceptive accuracy and reduce affective symptoms in individuals with autism (Quadt et al., 2021). While interoception has yet to be targeted directly during pregnancy, we know that similar practices that also engage interoception, such as mindfulness, reduce depressive symptoms in pregnant women (Matvienko-Sikar et al., 2016). Evidence supports the reduction of depressive symptoms in non-pregnant women via interoceptive modulation (de Jong et al., 2016), suggesting that behavioral and mindfulness interventions which reduce depressive symptoms during pregnancy may be partially mediated through alterations in interoceptive functioning. Future studies could test if interoceptive or mindfulness trainings are effective at improving interoception in pregnant women and if those changes contribute to a reduction in depressive symptoms.

In terms of pharmacological treatments for peripartum depression that may act on interoception, a new class of drugs approved to treat postpartum depression – zuranolone (oral) or brexanolone (intravenous infusion) – are of particular interest (Deligiannidis et al., 2023). These drugs have been shown to be effective in reducing depressive symptoms in the postpartum period and do so very rapidly (within hours to a few days, compared to several weeks for standardly prescribed antidepressants; Deligiannidis et al., 2021). Interestingly, zuranolone and brexanolone are allopregnanolone agonists, also targeting GABAergic neurons in the brain. As we reviewed, allopregnanolone and GABAergic neurons may be implicated in changes in interoceptive processing over the course of pregnancy. Therefore, it is possible that the beneficial effects of zuranolone and brexanolone in reducing postpartum depression, may also be realized through similar enhanced interoceptive mechanisms; a hypothesis which requires empirical investigation.

In addition to interventions targeted directly at interoception, there are other psychosocial factors that may underlie the maintenance of interoception and allostatic efficacy during this period of bio-psychosocial challenge. These include neural and cognitive flexibility, social support, and health habits (Callaghan et al., 2024). For example, humans can co-regulate allostasis through social interaction (Theriault et al., 2021). This provides opportunities for support figures to help offset the allostatic demands of pregnancy and the postpartum period, therefore buffering risk of depression. Future research should further explore each of these factors and their potential role in preventing postpartum depression via interoception and allostasis.

The framework presented here offers a novel understanding of peripartum depression by viewing pregnancy and the postpartum as sensitive periods for interoceptive functioning. While the mechanisms of this theory are supported by empirical work from various fields of psychology and neuroscience, additional work is needed to directly understand these relationships throughout the course of pregnancy and into the postpartum period.

## 9. Concluding remarks

In this perspective article, we present a novel framework to understand a mechanism that may underlie risk or resilience for peripartum depressive symptoms –interoceptive processing. We suggest pregnancy and the postpartum periods are characterized both by increased metabolic demands and a parallel increase in neuroplasticity. A key function that may benefit from this pregnancy/parenting-related neuroplasticity is interoception. Improved interoception can allow the brain to better model the metabolic needs of the body and in turn predict actions that maximize metabolic efficiency. Maintaining metabolic efficiency across pregnancy and into the postpartum can then lower the risk of depression for mothers. As biological underpinnings for this theory, we highlighted neural adaptations of pregnancy and postpartum, centered on interoceptive networks, paired with hormonal changes to progesterone and estrogen that may affect allopregnanolone and serotonin systems during pregnancy, and oxytocin during the postpartum. In turn, these effects may impact inhibitory signaling and E/I balance in the brain, serving to improve interoceptive functioning via higher precision weighting and precise prediction errors. This framework also provides further rationale for why mothers exposed to childhood adversity are at greater risk for depression during pregnancy and the postpartum and emphasizes why certain interventions such as mindfulness, interoceptive training, and neuro-steroids may be effective in preventing or treating peripartum depressive symptoms via changes in interoception. Ultimately, we hope this framework can be used to inspire new hypotheses and studies that can bring us closer to breakthrough discoveries in the prevention and treatment of peripartum depression.

## Figures and Tables

**Table 1:** Predictive processing key terms.

Term	Definition
Prediction	The anticipated sensory information generated by the brain based on prior experience and current context.
Prediction Error	The difference between a prediction and sensory information.
Prior(s)	The accumulation of previous experiences which are used to predict future sensory information.
Precision	The inverse variance of a prediction or prediction error.
Precision Weight	Neurons which alter the precision of prediction and prediction error signals based on the confidence in predictions or reliability of sensory data.
Allostasis	The process by which the brain makes predictions and prepares to meet the needs of the body before they arise.
Interoception	The process by which the brain perceives, integrates, and models sensory information generated from within the body.
Granular Laminar Structure	Granular regions are characterized by six well-distinguished laminar layers and tend to be close to sensory (interoceptive and exteroceptive) surfaces.
Agranular Laminar Structure	Agranular regions are characterized by poor differentiation between laminar layers, the lack of a layer IV, and are relatively further away from sensory surfaces.
